# Jervell and Lange-Nielsen Syndrome due to a Novel Compound Heterozygous *KCNQ1* Mutation in a Chinese Family

**DOI:** 10.1155/2020/3569359

**Published:** 2020-05-16

**Authors:** Yue Qiu, Sen Chen, Xia Wu, Wen-Juan Zhang, Wen Xie, Yuan Jin, Le Xie, Kai Xu, Xue Bai, Hui-Min Zhang, Xiao-Zhou Liu, Xiao-Hui Wang, Yu Sun, Wei-Jia Kong

**Affiliations:** ^1^Department of Otorhinolaryngology, Union Hospital, Tongji Medical College, Huazhong University of Science and Technology, Wuhan 430022, China; ^2^Department of Otorhinolaryngology, Distinct HealthCare, Wuhan, China; ^3^Institute of Otorhinolaryngology, Tongji Medical College, Huazhong University of Science and Technology, 430022 Wuhan, China

## Abstract

Jervell and Lange-Nielsen syndrome (JLNS) is a rare but severe autosomal recessive disease characterized by profound congenital deafness and a prolonged QTc interval (greater than 500 milliseconds) in the ECG waveforms. The prevalence of JLNS is about 1/1000000 to 1/200000 around the world. However, exceed 25% of JLNS patients suffered sudden cardiac death with kinds of triggers containing anesthesia. Approximately 90% of JLNS cases are caused by *KCNQ1* gene mutations. Here, using next-generation sequencing (NGS), we identified a compound heterozygosity for two mutations c.1741A>T (novel) and c.477+5G>A (known) in *KCNQ1* gene as the possible pathogenic cause of JLNS, which suggested a high risk of cardiac events in a deaf child. The hearing of this patient improved significantly with the help of cochlear implantation (CI). But life-threatening arrhythmias occurred with a trigger of anesthesia after the end of the CI surgery. Our findings extend the *KCNQ1* gene mutation spectrum and contribute to the management of deaf children diagnosed with JLNS for otolaryngologists (especially cochlear implant teams).

## 1. Introduction

Jervell and Lange-Nielsen syndrome (JLNS) is a rare autosomal recessive hereditary disorder characterized by profound congenital deafness and a prolonged QTc interval (greater than 500 milliseconds (msec)) in the ECG waveforms [1]. The prevalence of JLNS is about 1/1000000 to 1/200000 around the world [2]. Mutations of two genes, *KCNQ1* and *KCNE1* gene, are the causes of the disease. And approximately 90% of cases are due to *KCNQ1* gene mutations [1, 2]. Up to now, more than 550 mutations in the *KCNQ1* gene have been reported according to Human Gene Mutation Database (HGMD) (http://www.hgmd.cf.ac.uk/ac/index.php).

The *KCNQ1* gene, located on chromosome 11p15.5-p15.4, consists of 16 exons [3]. It encodes the *α*-subunit of a voltage-gated potassium ion channel (Kv7.1) [4]. The subunit contains six transmembrane segments (S1–S6), a pore-loop between S5 and S6 and two intracellular domains (N-terminus and C-terminus) [5]. The C-terminus contains a region (~100 amino acids) called A-domain. The A-domain consists of three subdomains (head, linker, and tail) and directs Kv7.1 to specifically assemble with KCNE *β*-subunits but not with other KCNQ *α*-subunits [6, 7]. The A-domain Tail is involved in proper channel trafficking and normal cell surface expression [6, 8]. The Kv7.1 has to co-assemble with a *β*-subunit of the potassium channel (IsK, encoded by *KCNE1* gene) to produce a slow delayed rectifier K^+^ current known as IKS, which is associated with regulation of potassium flow [4]. The *KCNQ1* and *KCNE1* gene are both expressed on the apical membranes of marginal cells of stria vascularis to form IKS, which contributes to the generation of endocochlear potential (EP) to maintain the inner ear potassium homeostasis [9, 10]. In addition, IKS is one of the repolarizing potassium currents in cardiovascular muscle cells that contribute to the cessation of the cardiac action potential and regulates cardiac action potential duration [9, 11]. Animal experiments showed that *kcnq1*^−/−^ mice exhibited deafness, vestibular dysfunction, and altered cardiac repolarization resembling patients with JLNS. Histological analysis showed collapsed Reissner's membrane, massive loss of hair cells as well as abnormal morphology of saccule, utricle, and semicircular ducts in *kcnq1*^−/−^ mice [12]. *kcnq1*^−/−^ mice with *kcnq1* gene replacement therapy in immature scala media by Lin et al. showed significantly improved hearing ability, normal cochlear morphology, and almost normal vestibular function which is optimistic for treatment of JLNS patients [11].

JLNS patients have a high incidence of sudden cardiac death (exceed 25%) [13]. However, this disease can occur among deaf children without obvious symptoms of cardiac events [13, 14]. Exercise, emotion, swimming, auditory stimuli, anesthesia, and fever have been reported as triggers of cardiac arrhythmias in children with JLNS [14, 15]. Even though the clinical diagnosis of this disease is straightforward, genetic evaluation using next-generation sequencing (NGS) is essential, because it has been reported that the patients with *KCNE1* gene mutations have a relatively lower risk of arrhythmic events than that with mutations of *KCNQ1* gene [16]. This influences the management of patients. Here, we report a deaf case diagnosed with JLNS in a Chinese family and a novel compound heterozygous mutation in *KCNQ1* gene associated with the disease.

## 2. Materials and Methods

### 2.1. Family Information

**Figure 1:**
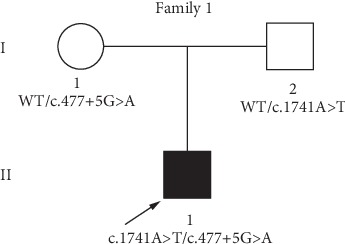
Pedigree of Family 1 associated with JLNS. A novel compound heterozygous mutation, c.1741A>T/c.477+5G>A was found in Family member 1-II-1. Family member 1-I-1 and Family member 1-I-2 were heterozygous carriers. The proband is shown in black and indicated by a black arrow. WT: wild type.

This Chinese family, named family 1, is associated with JLNS and contains three family members (son, mother, and father) (Figure 1). The proband 1-II-1 is three years and four months old. He passed the neonatal hearing screen (NHS). However, his parents gradually found he had hearing loss and cannot speak words. The child began wearing hearing aids at the age of one year and 8 months. However, it has no effect. Then, he underwent cochlear implantation (CI) surgery in the right ear when he was 2 years and 8 months old. The proband had the history of convulsion. His parents' hearing is normal.

### 2.2. Clinical Assessment

A series of audiological assessment was performed for the proband 1-II-1, which included otoscopic examination, conditioned play audiometry (CPA), auditory brainstem response (ABR), auditory steady-state evoked response (ASSR), auditory immittance, and distortion product otoacoustic emission (DPOAE). The child also underwent electrocardiography (ECG) and imaging tests (computed tomography, CT and magnetic resonance imaging, MRI). According to Jervell and Lange-Nielsen syndrome updated in 2017 GeneReviews, the diagnosis of JLNS is established with the profound congenital sensorineural deafness and long QTc interval (>500 msec). Identification of biallelic pathogenic variants in either KCNQ1 or KCNE1 confirms the diagnosis [1]. The World Health Organization (WHO, 1991) hearing impairment (HI) grade system includes five grades: no impairment, ≤25 dB nHL; mild, 26-40 dB nHL; moderate, 41-60 dB nHL; severe, 61-80 dB nHL; profound, ≥81 dB nHL; the audiometric dB nHL (International Standards Organization, ISO) values are averages of values at 500, 1000, 2000, and 4000 Hz for the better ear [17].

### 2.3. Genetic Tests

Written informed consent was obtained from the whole family. About 5 mL peripheral venous blood was collected from three family members for Deafness panel sequencing/NGS and Sanger sequencing which were performed by BGI Genomics (Wuhan, China). For the following experiments, the genomic DNA of blood samples was extracted according to the manufacturer's standard procedure of QIAamp DNA Blood Midi Kit (51185, Qiagen Inc., Valencia, CA, USA), then fragment the DNA by Covaris LE220 (Massachusetts, USA). The fragmented DNA (200-250 bp) was used to generate repair-end library according to Illumina protocols. Targeted DNA fragments were captured by SeqCap EZ Choice (NimbleGen, Madison, USA), followed by postcapture amplification. The products were sequenced on the BGISEQ-500 platform using BGISEQ-500RS High-throughput sequencing kit (PN: 85–05238-01, BGI). The SeqCap EZ Choice was designed to cover all exons together with the flanking exon and intron boundaries (±15 bp) of 127 known deafness-related nuclear genes and deafness-related mitochondrial regions. Postsequencing, a few unqualified sequences were removed from the primary data using a local dynamic programming algorithm. Then, the filtered clean reads were aligned to the Genome Reference Consortium Human genome build 37 (GRCh37)/Human genome build 19 (hg19) by the BWA (Burrows Wheeler Aligner) Multi-Vision software package. After alignment, the single-nucleotide variants (SNVs) and inserts and deletions (InDels) were called by SOAPsnp software and Samtools. All variants were further filtered and estimated via multiple databases including National Center for Biotechnology Information (NCBI) (https://www.ncbi.nlm.nih.gov), 1000 Genomes (http://phase3browser.1000genomes.org/index.html), Nucleotide Polymorphisms (dbSNP) (http://www.ncbi.nlm.nih.gov/projects/SNP/), HGMD (http://www.hgmd.cf.ac.uk/ac/index.php). Pathogenic variants are assessed under the American College of Medical Genetics and Genomics–Association for Molecular Pathology (ACMG–AMP) guideline. Filtered candidate variants were confirmed by conventional Sanger sequencing methods. The methods we used have previously been published [18]. The complete nucleotide and amino acid sequence of the *KCNQ1* gene is shown in the NCBI (https://www.ncbi.nlm.nih.gov/gene/).

## 3. Results

### 3.1. Clinical Data

**Figure 2:**
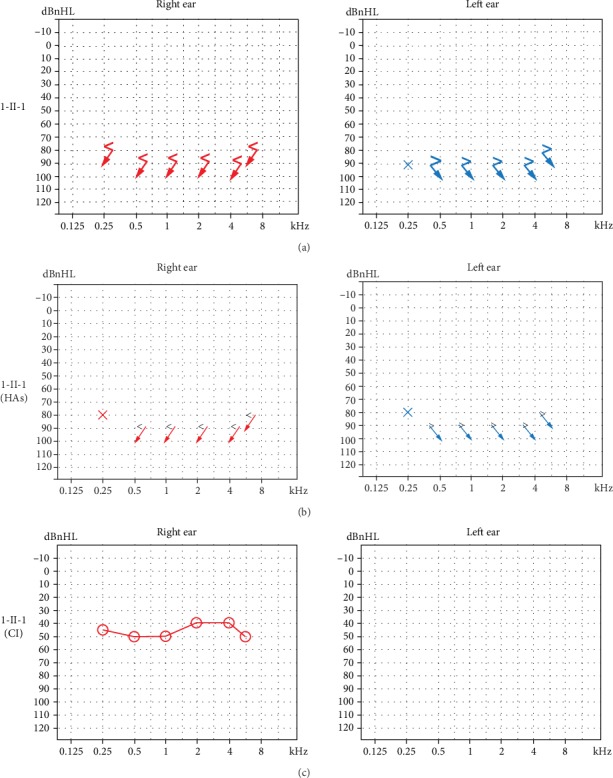
CPA results for proband 1-II-1 before and after wearing hearing aids (HAs) and with cochlear implant (CI). (a) Testing results before wearing hearing aids. (b) Testing results after fitting of hearing aids. (c) Sound field thresholds of the right ear 3 months after CI surgery. Arrows: no response at the specific frequency. Cross and circle: threshold at the specific frequency.

**Figure 3:**
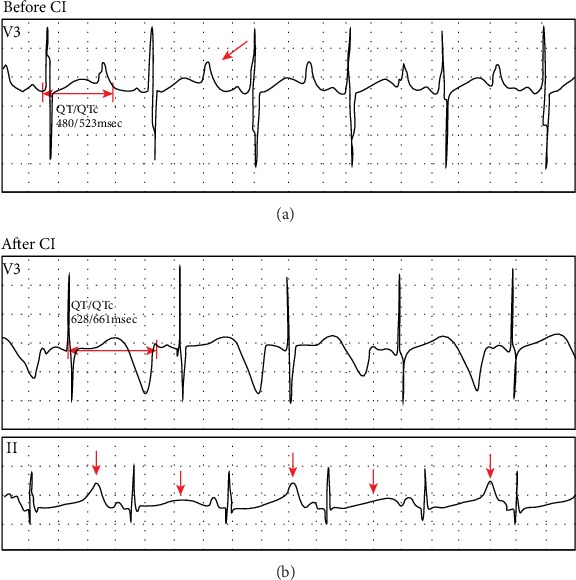
ECG detected in Proband 1-II-1. (a) The ECG waveforms before cochlear implantation (CI). Two-way arrow: QT/QTc interval. One-way arrow: dome and dart T wave. (b) The ECG waveforms after cochlear implantation (CI). Two-way arrow: QT/QTc interval. One-way arrows and arrowheads indicate T wave alternans (TWA).

The testing results for proband 1-II-1 were as follows. CPA showed a response of 90 dB nHL at 250 Hz (left ear) and no response of both ears in the remaining frequencies (Figure 2(a)). Bilateral tympanograms were type A. No wave of ABR can be elicited at 105 dB nHL bilaterally (data not shown). DPOAEs were absent in both ears (data not shown). The thresholds of ASSR were 100 dB nHL at 500 Hz, 100 dB nHL at 1 KHz (left ear), and 90 dB nHL at 500 Hz, 95 dB nHL at 1KHz (right ear). After hearing aids were fitted, CPA revealed response of 80 dB nHL at 250 Hz of both ears (Figure 2(b)). The CI was implanted into the right temporal bone. Three months after CI surgery, average of audiometric values of all frequencies was 45.8 dB nHL (Figure 2(c)). ECG detected a prolonged QT interval (QT/QTc: 480/523 msec), dome and dart T waves in V3 before CI surgery (Figure 3(a)). After end of the surgery, ECG exhibited T wave change and T wave alternans (TWA), and the QT/QTc interval is 628/661 msec (Figure 3(b)). Imaging tests (CT and MRI) showed no abnormalities.

### 3.2. Variants Identification and Analysis

**Figure 4:**
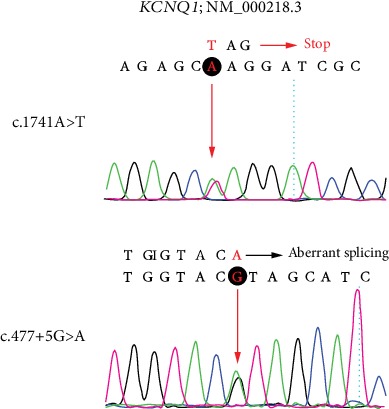
Mutated *KCNQ1* sequences of the identified c.1741A>T (above) and c.477+5G>A (below) variant. The mutated nucleotide is shown in red. Red “stop” indicates termination of synthesis. I: boundary of corresponding exon and intron. Red arrows and black rounds: sites of nucleotide changes.

**Figure 5:**
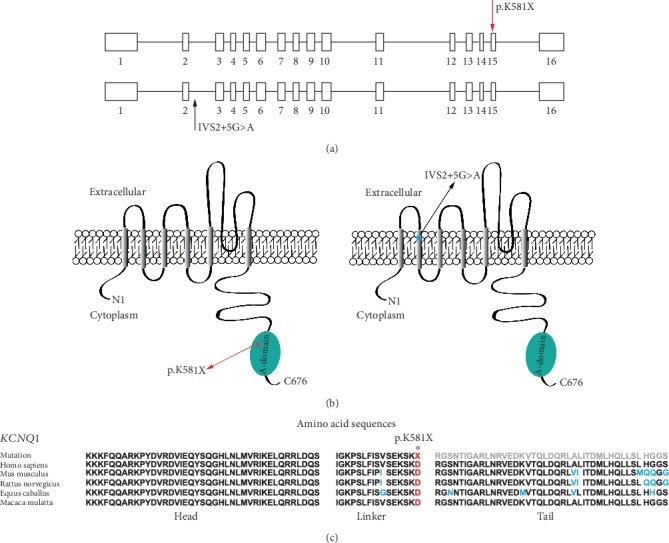
Molecular basis for the case of JLNS is detailed at gene and protein levels and evolutionary conservation of amino acids in A-domain affected by the nonsense mutation. (a) Schematic diagram of 16 exons and 15 introns encoded by biallelic *KCNQ1* genes (*KCNQ1*; NM 000218.3). Two variants were indicated by arrows. Novel mutation is shown in red and known mutation is shown in black. Rectangle: exon. Line: intron. (b) Schematic diagram of *α*-subunit of IKS encoded by biallelic *KCNQ1* genes (*KCNQ1*; NM_000218.3) with pathogenic mutations of p.K581X (red cross) or IVS2+5G>A (blue rectangle). Six transmembrane segments (S1-S6) are indicated by gray columns and the pore-loop is located between S5 and S6. Green oval: A-domain. Red arrow: novel mutation. Black arrow: previously-reported mutation. (c) Evolutionary conservation of A-domain (head, linker, and tail). Mutated site is indicated by asterisk. Gray residues cannot be translated. Different residues were indicated in blue.

Using deafness panel sequencing, we excluded *KCNE1* gene mutation which was identified as the cause of JLNS and found a novel compound heterozygous mutation c.1741A>T/c.477+5G>A in *KCNQ1* gene of proband 1-II-1 (Figure 4). The c.1741A>T (p.K581X) was a novel mutation, and the c.477+5G>A (IVS2+5G>A) has been reported [15, 19]. The mutation c.1741A>T was a nonsense mutation with substitution of no. 1741 nucleotide from adenine to thymine (Figure 4). It occurred in exon 15 (Figure 5). The c.477+5G>A occurred in intron 2 with a mutation of no. 477+5 nucleotide from guanine to adenine (Figures 4 and 5). It was a splice mutation. Both mutations were not polymorphic sites, and the prevalence of them was 0 in 1000 Genomes. The mutation p.K581X was not listed in dbSNP and has not been reported before. The IVS2+5G>A has been recorded in HGMD [15], and its RS number in dbSNP was rs397508111. The parents of the child were both heterozygous carriers (Figure 1). According to the 2015 American College of Medical Genetics and Genomics–Association for Molecular Pathology (ACMG–AMP) guidelines [20] and its refinement in 2017 [21], the variant c.1741A>T (p.K581X) was likely pathogenic, and the variant c.477+5G>A (IVS2+5G>A) was pathogenic.

## 4. Discussion

A novel compound heterozygous mutation c.1741A>T/c.477+5G>A in *KCNQ1* gene was found in the proband 1-II-1with JLNS. Combining the medical history and results of audiological examinations, family member 1-II-1 was diagnosed with profound congenital sensorineural deafness according to the 2018 international consensus (ICON) on audiological assessment of hearing loss in children [22] and the WHO-HI grade system. The child passed the NHS which was an OAE-based test; we speculated that it was because the function of hair cells was not affected at birth. It was reported by Casimiro et al. that *kcnq1*^−/−^ mice exhibited the normal hair cell morphology at birth and delayed hair cell loss [12]. Normal QTc interval in males is <440 msec [1]. ECG detected a prolonged QTc interval (523 msec) in the patient before cochlear implantation (Figure 3). Therefore, the proband 1-II-1 was diagnosed with JLNS according to the diagnostic criteria above. It has been reported that mutations of *KCNQ1* and *KCNE1* gene were the causes of JLNS [1, 2]. We used NGS+Sanger sequencing to identify the genotype of the patient. Genetic testing results showed the *KCNE1* gene mutation was excluded. And two mutations in *KCNQ1* gene, c.1741A>T (p.K581X) and c.477+5G>A (IVS2+5G>A) were identified in the family (Figure 1). The variant p.K581X was novel, while the other one IVS2+5G>A was known previously. The IVS2+5G>A had been reported in individuals affected with LQTS or prolonged QT intervals [15, 23, 24]. And this variant had also been identified to be compound heterozygous with p.Y171X in a patient with JLNS [19]. Research on assessment of variant of unknown significance in LQTS revealed that IVS2+5G>A carrier had a prolonged end-recovery QTc interval underwent an exercise stress [25]. Millat et al. have raised a “double-dose” effect that multiple or compound gene mutations occurring in LQTS families may result in a more severe clinical phenotype [23]. This can explain that individuals shared the same single mutation IVS2+5G>A but with varying degree symptoms (no symptoms, only with a prolonged QT intervals, with cardiac events, with profound deafness [15, 19, 23, 25]). According to the ACMG–AMP criteria and its refinement, the variant IVS2+5G>A was pathogenic. Here, we found IVS2+5G>A was compound heterozygous with p.K581X in proband 1-II-1 (Figure 1). The amino acid residues at the novel mutation site (p.K581X) were highly conserved across different species (Figure 5(c)). Through filtration and estimation via multiple databases, the mutation p.K581X was not a polymorphic site and not listed in 1000 Genomes. The p.K581X was judged as a likely pathogenic variant by ACMG criteria. And the parents of Proband 1-II-1 were both heterozygous carriers without deafness (Figure 1). These all suggested that the compound heterozygous mutation p.K581X/IVS2+5G>A may have a “double-dose” effect contribute to pathogenesis of JLNS.

*KCNQ1* gene mutations may impair the structural and/or functional ion channel (Kv7.1) resulting in dysfunction of IKS, abnormal regulation of potassium flow, and the onset of JLNS. IVS2+5G>A occurred in consensus splice site of intron 2 with the nucleotide substitution from guanine to adenine (Figures 4 and 5(a)). Nucleotide substitutions within the consensus splice site are relatively common causes of aberrant splicing [26]. And a study on the mutation pattern of aberrant 5'splice sites revealed that the point mutations at the position +5 were particularly prone to aberrant splicing [27]. Experiments to evaluate the significance of consensus splice sequence in splicing have shown that mutation at the +5 position of the exon 2 of the rabbit *β*-globin gene disturbed correct splicing and resulted in joining of exon 1 to exon 3 [28]. The variant p.K581X occurred in exon 15 leading to premature termination of peptide synthesis. This nonsense mutation may result in no protein synthesis or synthesizing truncation proteins. Researches have revealed that mutations in A-domain impaired the ability of the channel to reach the plasma membrane. And the integrity of the A-domain Tail is critical for normal cell surface expression of Kv7.1 [6, 8]. The variant p.K581X occurred in A-domain Linker which may synthesize a truncation protein without A-domain Tail (Figure 5). Amino acid sequence analysis showed the residues of the region containing the novel mutation site were highly conserved in Homo sapiens, Mus musculus, Rattus norvegicus, *Equus caballus*, and Macaca mulatta (Figure 5(c)). Therefore, we speculated that p.K581X resulted in affecting normal cell surface expression of Kv7.1 leading to dysfunction of IKS and impaired regulation of potassium flow in the heart and inner ear. Profound hearing loss is one of the clinical phenotypes in all JLNS patients. It was confused that *KCNQ1* gene mutation resulted in such a severe phenotype. Besides affected EP, *kcnq1*^−/−^ mice showed massive loss of hair cells [12]. As EP reduction not always lead to profound deafness [29], hair cell loss in *kcnq1*^−/−^ mice should be noticed. Degeneration of hair cells was observed in most deafness mice models induced by gene mutations, ototoxic drugs, and noise [30–33]. And lots of experiments on regeneration hair cells have been carried out [34, 35], which may be a new strategy to improve the hearing ability of JLNS patients. It is not clear why the knockout *kcnq1* gene resulted in hair cell loss. Apoptosis or autophagy of hair cells has been observed in kinds of deafness mice models [36–38], which may be involved in the degeneration of hair cells in *kcnq1*^−/−^ mice. Next, we need to conduct experiments to verify the dysfunction of Kv7.1 induced by *KCNQ1* gene mutations and explore the mechanism of deafness.

Cochlear implant (CI) is beneficial to improving the hearing ability of JLNS patients. A review of literature about the outcome of CI in these patients confirms good auditory outcome with their devices [13]. Because of no effect of wearing hearing aids and good auditory outcome with CI, proband 1-II-1 successfully underwent a CI surgery in the right ear and acquired good ability of hearing (Figure 2). With the mapping of CI, the child exhibited satisfactory auditory outcome in daily life. However, after the end of the surgery, the child had a convulsion and life-threatening cardiac arrhythmias (Figure 3). With propranolol treatment and accurate management in pediatric intensive care unit (PICU), no cardiac events occurred. As anesthesia has been identified as a trigger of cardiac arrhythmias in JLNS patients [13], careful attentions should be given at the induction of anesthesia, during wake up and after the surgery. It suggested that JLNS patients should be equipped with paddles for defibrillation during surgery for being exposed to ventricular arrhythmias [14]. The genotype of the proband 1-II-1 showed a compound heterozygous mutation in *KCNQ1* gene. According to Schwartz et al., JLNS patients with *KCNQ1* gene mutations had six-fold greater risk of arrhythmic events than that with mutations in *KCNE1* gene [16]. These all warned that otolaryngologists (especially cochlear implant teams) should be aware of the risk of the disease and take precautions dealing with the deaf children diagnosed with JLNS.

## 5. Conclusions

As mentioned above, we found a novel compound heterozygous *KCNQ1* gene mutation (c.1741A>T/c.477+5G>A) associated with JLNS, which suggested a high risk of cardiac events in our patient. In the process of management, the child had a good outcome with CI. However, life-threatening arrhythmias occurred with a trigger of anesthesia after the end of surgery. This warned that otolaryngologists (especially cochlear implant teams) should be aware of the hazards and take precautions dealing with the deaf children diagnosed with JLNS. Our findings extend the *KCNQ1* mutation spectrum and contribute to the management of deaf patients who are diagnosed with JLNS.

## Data Availability

The data which support the conclusions of our study is included within the article.
